# Acoustic Properties of Resonant Spruce Wood Modified Using Oil-Heat Treatment (OHT)

**DOI:** 10.3390/ma13081962

**Published:** 2020-04-22

**Authors:** Przemysław Mania, Mateusz Gąsiorek

**Affiliations:** Department of Wood Science and Thermal Technics, Faculty of Wood Technology, Poznań University of Life Sciences, Wojska Polskiego 38/42, 60-627 Poznań, Poland; mateus.gasiorek@gmail.com

**Keywords:** spruce wood, resonant wood, oil-heat treatment, acoustic properties, sound velocity

## Abstract

Wedge-shaped boards of spruce wood (*Picea abies* Karst.) are used to make violin fronts, also known as soundboards. Oil-heat treatment (OHT) can influence the acoustic properties of resonant wood, such as spruce. In this study, the effect of OHT on spruce wood was evaluated, using palm oil as a heating medium, at four different temperatures: 140, 160, 180 and 200 °C. Physical, mechanical and acoustic properties of spruce wood were evaluated before and after OHT and included the following: density, modulus of elasticity in the static bending test, and wood sound velocity. The acoustic parameters after OHT improved; however, the samples bent after modification had a higher modulus of elasticity, with a simultaneous deterioration of the acoustic parameters. The dynamic modulus of elasticity increased by 11%, and the musical constant by 5%. The static modulus increased by more than 3.5%, but the acoustic parameters calculated on the basis of these results indicated a deterioration of the acoustic properties of completely oven-dried wood. The increase in moisture content to air-dried condition contributed to a slight increase in the mean musical constant at the highest modification temperature.

## 1. Introduction

The suitability of wood for the construction of musical instruments depends on acoustic parameters, with modulus of elasticity (MOE) and wood density being the primary properties. The main acoustic parameters are speed of sound (v), acoustic resistance (z) or sound absorption coefficient (α) [1]. In order to modify these parameters, wood can be subject to modification. There are several types of modification processes: chemical methods, thermal methods, surface modification and impregnation. For example, thermal modification of many types of wood is associated with a decreased density. It is also possible, for relatively mild processes, to increase the MOE and hardness [2,3,4,5]. Thermal modification of wood, in addition to changing its density and MOE, may change other properties. At the beginning of the 20th century, Tiemann found that drying wood at high temperatures reduces its equilibrium moisture content [6]. One effect of this action is a greater biological durability of wood [7,8,9,10] and increased dimensional stability [11,12,13,14].

OHT (oil-heat treatment) uses vegetable oil as a heating medium, which has two purposes: (1) quick and even transfer of temperature to the wood over its entire surface; and (2) prevention of oxygen from reaching the wood during modification [15]. The oils used (palm, linseed, rapeseed, sunflower, coconut and sunflower) are nontoxic, and their low price reduces the cost of this process [16]. Modification time depends on the dimensions of the wood to be modified and should be sufficient for the temperature in the center to reach the required modification temperature. Oil-heat-treated wood, despite the absorption of oil, does not pose a problem when gluing or varnishing [15]. This is in line with findings that vegetable oils do not penetrate cell walls [17], making it possible to apply a coat of paint. However, it is not recommended to use wood after modification without any coating for outdoor use, as it is not weather-resistant [18].

OHT leads to a very strong reduction of the fiber saturation point level, i.e., up to 14% [15]. This is also related to the increased dimensional stability of the wood [11]. Studies have shown that wood modified in vegetable oil is of better quality than wood modified in a gaseous atmosphere; changes resulting from OHT are more advantageous than in the case of heated gas modifications [19]. For example, after hot-oil modification, the MOE in wood is about 10% higher than after air modification at the same temperature [15,17]. One unfavorable effect of OHT is an increase in the weight of wood due to absorption of small amounts of oil [17]. Absorption occurs when the oil temperature drops after the modification with samples still in the medium, or when the material is extracted from the oil immediately after the modification time. Studies have also shown changes of resonance parameters of wood after biological modification [20] and descriptions of changes in the mechanical parameters of wood after thermal modification in a superheated steam atmosphere [8,14,21,22,23,24]. However, to date, we have found no study describing the direct impact of OHT modification on the acoustic parameters of resonant wood. Given the above, the purpose of this study was to determine the effect of the oil-heat treatment of good-quality resonant spruce wood on acoustical parameters, especially acoustic constant, sound velocity and acoustic impedance.

## 2. Materials and Methods

A total of 15 violin wedges, intended for the construction of the top plates of the violin resonant body, were used in this study. Wedges made of spruce wood (*Picea abies* Karst.) were obtained from the mountain areas of the Silesian Beskid (near the village of Istebna). The material was free from defects in any form and had been seasoned for at least 5 years, under constant environmental conditions (T = 20 °C and RH = 50% ± 2%). Wedges with minimum dimensions of length 480 mm, width 180 mm in the radial direction and thickness 10–60 mm in the tangential direction were characterized by a straight grain arrangement. A total of 130 samples with dimensions of 10 mm × 10 mm × 150 mm (last dimension along the grain) were obtained from these wedges, for the static bend test.

Density of wood (*ρ*) was determined on each wood sample, designed to determine the bending strength of wood. The sample densities were determined according to the method recommended by ISO 13061-2:2014 [25]. The mass of each sample was measured on an analytical balance (Sartorius GmbH, Göttingen, Germany) (±0.001 g accuracy). The dimensions were measured with a digital caliper, to the accuracy of ±0.01 mm.

Mechanical tests were made by using the equipment ZWICK ZO50TH wood testing machine (Zwick/Roell, Ulm, Germany), which software allowed to calculate, e.g., MOE and modulus of rupture (MOR). The three-point bending test was carried out in accordance with PN-77/D-04103 [26]. The determination of MOE on each sample was preceded by the determining a value of the proportional limit force for samples of different density. For this purpose, 16 samples with a different macrostructure were subjected to a bend test for destruction. In this way, the mean value of the stress and force at the proportional limit was determined, which was 200 N. The bending of the samples with a force less than the proportional limit does not cause permanent deformation in the wood, so it was possible to reuse the beams for measuring the MOE, after the OHT modification. To determine the MOE, the sample was placed on 120 mm spacing supports of a ZwickRoell testing machine (Zwick/Roell, Ulm, Germany). The direction of the bending force was tangential to the annual increments. The moisture content of wood has a very strong influence on the mechanical properties of wood [27]. To eliminate this factor in the wood before and after modification, the determination of MOE was carried out at a wood moisture content close to MC = 0%. This determines the direct impact of thermal modification on mechanical and acoustic parameters. Next, analogous determinations were carried out after air-conditioning of the samples at T = 20 °C and humidity RH = ~65%, which allowed us to achieve equilibrium moisture content of the control wood, at MC = 9%, which allowed us to check how the wood parameters change under normal environmental conditions.

After determining the density and mechanical parameters of all samples, they were divided into 4 groups (of similar mean values), for modification under different temperature variants. Modification was carried out in 4 temperature variants: 140, 160, 180 and 200 °C. These are the temperatures used when modifying wood for testing [14,21], and in industry [15]. By using several variants of the modification temperature, it was possible to determine how the temperature affects the properties of the wood. Modification was carried out by using popular and frequently used palm oil. Samples were placed in the heated oil, in a tripod, ensuring free flow of medium to each sample wall. The modification time was measured from the moment the analyzed modification temperature was reached. On the basis of data on the dynamics of wood heating [15] and preliminary studies with thermocouples, a modification time of 4 h was assumed for small samples, while for full wedges it was 16 h. After the designated modification time, the samples were taken out of the oil immediately; their surfaces were dried, to minimize the amount of oil absorbed by the samples, and then they were placed in a desiccator, to cool down and keep dry. The MOE was then determined in oven-dried and air-dried conditions. Five whole resonance wedges were modified, to determine whether the size of the modified element affects the obtained results. Large elements were modified only at the highest temperature, i.e., 200 °C. The speed-of-sound propagation in wood was determined on these samples.

On the whole resonance wedges, the speed-of-sound propagation in radial and longitudinal direction and the sonic MOE were determined. The determination of the speed-of-sound propagation was made by measuring the time of passage of the wave through the tested sample. The transmission time of the waveform through the tested sample was measured, using the material sampler type 543E (UNIPAN, Warsaw, Poland), equipped with transmitting and receiving heads of 1 MHz frequency. A minimum of 10 measurements of the time of passage of the sound wave in the radial direction and 4–6 in the longitudinal direction were taken on each sample. The speed of the sound wave was calculated (with an accuracy of 10 m·s^−1^), using the following formula:

(1) $$v=\frac{L^{R,L}}{t}\;(m\;\times \;s^{-1}),$$

where *L^R,L^* is sample size, respectively, radial (*R*) and along the grain (*L*) (m); and *t* is the propagation time (s).

Knowing the speed-of-sound propagation in wood and its density, the sonic MOE (MOE_D_), otherwise known as dynamic MOE, was calculated for the longitudinal direction only.

(2) $${\mathrm{MOE}}_{D}=v^{2}\times \rho \;(\mathrm{MPa}),$$

Basic acoustic parameters were calculated on the basis of the relevant physical parameters. Acoustic impedance was defined as follows:

(3) $$z=c\times \rho =\rho \times \sqrt{\frac{\mathrm{MOE}}{\rho }}=\sqrt{\mathrm{MOE}\times \rho }\;(\mathrm{kg}\;\times \;m^{-2}\;\times \;s^{-1}),$$

The musical constant, called acoustic constant, was defined as follows:

(4) $$A=\sqrt{\frac{\mathrm{MOE}}{\rho^{3}}}\;(m^{4}\times \;{\mathrm{kg}}^{-1}\;\times \;s^{-1}).$$

The experimental data were analyzed by using the DellTMStatisticaTM13.3 software (TIBCO Software Inc., Palo Alto, CA, USA), with the analysis of variance (ANOVA). Significant differences between mean values of the parameters describing the properties of spruce wood samples were determined by using Turkeys’ honest significant difference (HSD) test. Comparisons were considered significant at *p* ≤ 0.05.

## 3. Results and Discussion

Sample densities before and after modification are shown in Table 1.

The range of density change, Δ*ρ*, was calculated as follows:

$$\Delta \rho =\frac{\rho_{OHT}-\rho }{\rho }\times 100$$

The mean density of wood samples before modification was 437 kg × m^−3^. This allows the used material to be classified as good tonewood [28]. Mean values of the density of spruce music wood similar to those reported here were described previously by Buksnowitz et al. [29,30], studying wood for the production of musical instruments, and characterized by wavy annual increments (hazel growth). The density of the OHT samples increased in each variant according to temperature. The increase was between 7% and 10%. A slight increase in density, on average by 35 kg × m^−3^, was not statistically significant (ANOVA) for all temperature variants. The increase in wood density is mainly due to the increase in wood weight. During OHT thermal modification, mainly during the extraction of samples from the oil, a surface absorption of the heating medium occurs. An increase in mass therefore contributes to an increase in density. This increase in resonant spruce wood, compared to other wood species described in the literature, is not so high. In pine trees, it may exceed 18% [8]. However, spruce belongs to the species which are difficult to saturate; therefore, the increase in density was much lower, as it was difficult for the oil to penetrate deeper into the samples. However, a tendency can be observed: The higher the modification temperature is, the lower the relative increase in wood density. Lower density increases at higher modification temperatures may cause higher weight loss in these samples. Mass loss as a result of thermal modification is related to evaporation of side components of wood, such as terpenes, fat, wax and phenols that evaporate, and the products of decomposition of the least stable hemicelluloses. The density of wood increases with an increase in moisture content (MC). This increase after modification was much smaller than in oven-dried wood, and it was about 3.7%. However, similar increases, of about 4%, were observed for modification temperatures of 140, 160 and 180 °C. The smallest increase in density, approximately 2.7%, was observed for modification at 200 °C. This is due to thermal modification, which reduces wood hygroscopicity. The reduced ability to attach water molecules results in a smaller increase in mass and, consequently, in density.

Table 2 shows the MOE determined along the grains for the wood, before (MOE) and after thermal treatment (MOE_OHT_), at different temperatures and moisture-content levels.

The MOE for each modification variant was found to increase, on average, by 3.7%, in oven-dried wood, and by almost 9% following air-drying. MOE values have been reported to increase by up to 15% [8]. In our study, the mean value in the oven-dried state before modification was around 11.6 GPa, and the modification process increased the MOE to around 12 GPa, but these did not differ significantly. In line with this, Sailer et al. [31] found no differences in the MOE for oil-treated wood at 180, 200 and 220 °C. ANOVA did not reveal significant differences between the results of the MOE before and after modification of oven-dried wood. The minor increase in MOE after modification appear to be due primarily to increased wood density. The surface layers of wood have a higher density, as only these layers directly absorb part of the oil when they are removed from the bath. An increase in the MC of the samples results in a decrease in mechanical properties. In the bending test, strength can be reduced by about 50% when the moisture content of the wood increases from a completely dry state to the fiber saturation point [27]. The MOE is reduced by about 35%. In the case of the samples analyzed, the increase in MC to the air-dried state contributed to a decrease in MOE values to approximately 10.4 GPa in the control wood and to 11.3 GPa in the modified wood. The decreased MOE related to the change in wood moisture content was therefore 10.3% and only 5.8%, respectively. This shows the influence of thermal modification on the mechanical properties of wood. Reduced hygroscopicity, which is the result of thermal modification, has a positive effect on the resonance properties of spruce wood. As a result of thermal treatment of wood, its physical properties change, e.g., dimensional stability increases by up to 50%, and its hygroscopicity and absorbability decreases [6,32,33]. Samples conditioned under analogous laboratory conditions (φ = 55%, T = 20 °C) reached different levels of equilibrium moisture content. As a result of OHT modification, the mean MOE in resonant spruce wood increase by approximately 9%, with the highest increase occurring at the highest modification temperatures. However, the analysis of variance did not show statistically significant differences between these results.

The measure of the resistance, which is placed by particles of a given material against the wave energy, to induce them into an oscillatory motion, is the so-called acoustic impedance (z). Like the speed of sound, the characteristic impedance is directly related to the MOE and density of a material. Mean results for particular variants of modification temperature and moisture content are presented in Table 3. With high acoustic resistance of, e.g., steel, the vibration intensity of the particles of this medium is low [34]. Therefore, the energy of elastic motion consumption to set the particles in motion is low. In this case, most of this energy is passed through, and the sound wave passing through such a medium is slightly suppressed. At low acoustic impedance, particles are put in strong natural vibrations, which absorb a large part of the wave energy, which in turn causes significant damping. The wood should have the lowest possible acoustic impedance values.

The characteristic impedance of the wedge is an important parameter, since vibratory energy is transmitted from the string to the soundboard [35,36]. The mean results of our study do not largely differ from those reported by other investigators. Some authors have characterized acoustic impedance for resonant spruce wood in the range of 23.90–25.00 (kg × m^−2^ × s^−1^) [37,38]. OHT modification can contribute to increased acoustic impedance. For example, in oven-dried wood, it was about 5.8%, whereas in air-dried wood, it was about 6.3%. The increase in wood MC contributed to proportionally similar changes in both MOE and wood density, resulting in a very similar acoustic impedance value. There were no statistically significant differences between the results before and after thermal modification. This slight increase in acoustic impedance values indicates that spruce wood has no obvious drawbacks when used for the production of violin top plates. The aim is to minimize the differences between acoustic impedance of wood and air to about 429 kg × m^−2^ × s^−1^.

To determine which temperature used in the OHT modification process is most suitable for resonant wood (intended for the surface plates of stringed instruments), changes in wood density also need to be considered. The acoustic constant (A) was calculated, to compare the two values (oven-dried and air-dried) for each temperature. The values for the individual groups are shown in Table 4.

The musical constant in oven-dried wood was an average of 11.84. The wood tested here had good acoustic properties compared to those found in previous studies [39,40,41]. The musical constant, as shown in Table 4, decreased after thermal modification. For oven-dried wood, the reduction was between 8.1% and 11.4%. The decreased acoustic constant values, after modification, were caused by a relatively higher increase in mass (and thus density) compared to MOE. Increased moisture content was associated with decreased acoustic constant values. However, at this level of wood moisture, the differences between the different modification variants are negligible. For modification temperatures between 140 and 180 °C, the musical constant decreased by 1.2% to 2.2%, while at the highest modification temperature, it increased slightly (0.5%). Modification in hot oil as a heating medium can therefore lead to improved acoustic performance. The moisture content of individual elements of musical instruments made of wood usually corresponds to the conditions in which the instrument is used. Most often it is an MC of about 6%–10%. However, the oil should not be absorbed deep into the sample, and samples of larger volume should be modified so that the top layer is then grounded off. As mentioned above, spruce is a species that is difficult to saturate, since oil absorption tends to occur at the sample surfaces. In samples with a cross-section of 10 mm × 10 mm, relatively deep absorption occurred. After cutting the samples across the grain, the absorbed oil was in the surface layer up to 2 mm deep. For this reason, after modification of the entire resonance wedges in hot oil, at 200 °C, and determination of the density and time of sound propagation in each of them, about 3 mm of the material was planed, and the wedge fronts were cut off. As a result, the oil had no effect on the results during the next density determination.

**Figure 1:**
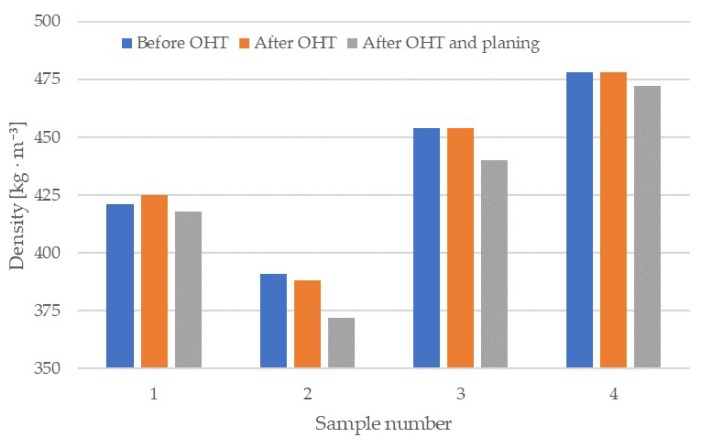
Wood density of the wedges in the different stages of the study.

The density of individual wedges, at different stages of the study, is shown in Figure 1. After modification, the increase in density occurred to a small extent, whereas after planing all wedge surfaces, the density was lower than initial values. This supports the finding that weight loss occurs during thermal modification in hot oil, as in other types of thermal modification [17].

The change of wood density after modification of the entire wedge was small and only found in samples 1 and 2; the difference in the first sample was 1%, and in the second 0.7%. The lowest density and the highest drop in density occurred in sample 2. As can be seen in Figure 1, for this sample, there is so much weight loss that the reduction in density occurs immediately after modification. It is also worth noting very similar density values before and after modification for samples 3 and 4 (density differences do not exceed tenths of kg × m^−3^). These results show how important the size of the modified element is for its subsequent parameters.

**Figure 2:**
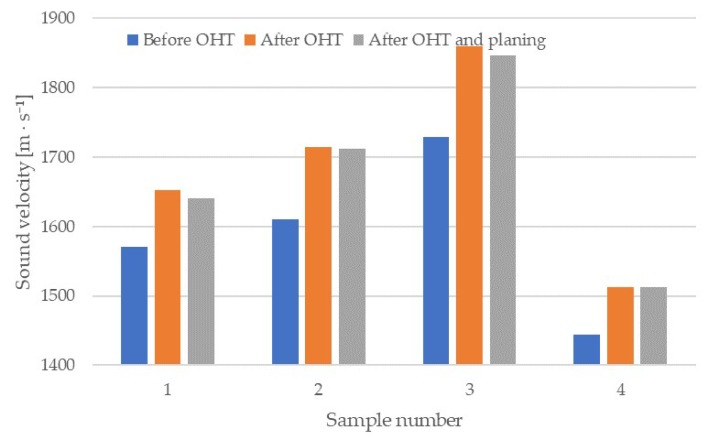
Speed-of-sound propagation in the radial direction of the wedges analyzed in the different stages of the study.

Using the same samples, we determined the speed-of-sound propagation in the radial direction, before and after modification. These measurements were then repeated after 3 mm of wood was ground off. Mean results obtained during this determination are shown in the graph in Figure 2. Means were calculated from 10 measurements taken on each sample.

The mean value of sound speed was 1590 m × s^−1^ before modification. The highest value was in sample 3 (1730 m × s^−1^), and the lowest in sample 4 (1440 m × s^−1^). Reported values for spruce music wood range widely from 1070 m × s^−1^ [42] to almost 1900 m × s^−1^ [30]. The process of OHT modification contributed to an increased speed-of-sound propagation in the wood, both immediately after the modification and after grinding off the layer where the oil was absorbed. The highest increase in the value of this parameter was observed in sample 3, at about 7.6%; the lowest was in sample 4, at about 4.8%. However, the ANOVA did not show statistically significant differences between the results before and after modification.

**Figure 3:**
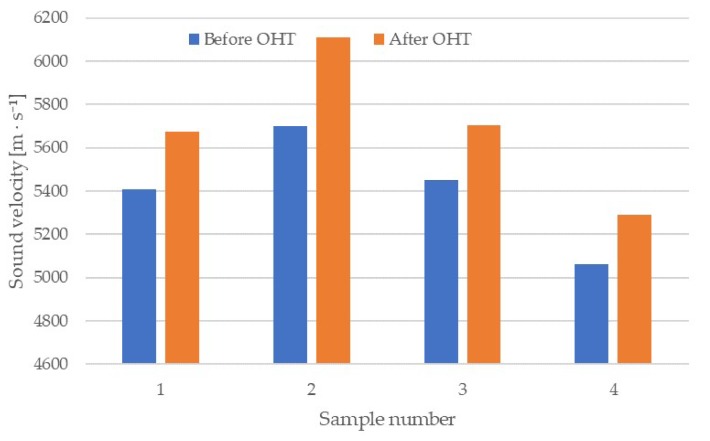
Speed-of-sound propagation in the longitudinal direction of the analyzed wedges, before and after modification.

Data for the speed-of-sound propagation along the grain are shown in Figure 3. This figure shows mean sound velocity in wood calculated from the results of 4–6 points on a given sample, made before and after modification. The process of cutting off the sample ends had no effect; therefore, data after planning were not included. In the longitudinal direction, modification also contributed to an increase in the speed of sound in the wood. The mean speed before modification was 5405 m × s^−1^, and after was 5695 m × s^−1^. This increase was 5.6%, and with a notably higher value in sample 2, almost 7.6%. The speed of sound in the resonant spruce wood was similar to the values described by others, since these values can range from 5200 to 6000 m × s^−1^, according to Holz [40] and Bucur et al. [43]. In sample 2, the difference between the mean speed of sound before and after OHT modification was statistically significant.

Based on the speed-of-sound propagation in wood, the values of sonic MOE were calculated (see Table 5).

The mean value of sonic MOE for the analyzed samples was higher than the static modulus, around 12.7 GPa (Table 2). OHT modification contributed to a marked increase in this value, as it averages 11% higher. The highest increase was found in sample 2, and the lowest was found in sample 4. It was a much higher increase than that observed in the static module (Table 2). The differences were statistically significant for all samples.

Using the data for whole wedges (wood density and dynamic MOE), we calculated the values of acoustic constant. The results are shown in Table 5. These analyses demonstrate that OHT modification does not modify wood properties in a uniform way. For samples which were bent (both oven-dried and air-dried), MOE was found to decrease after OHT. In contrast, for larger elements, e.g., violin wedges, this value increases, on average, by 5.3% after OHT, as shown in Table 5. It follows that the modification of resonant spruce wood has no consistent impact on the acoustic properties of the wood. There is an increase in sound resistance, which, in the case of a resonant body, means that more energy has to be transferred from the strings to the body, to generate vibrations. However, there is an improvement in the speed of sound in the wood, resulting in faster wave propagation through the elements of the body and also an increase in the damping value by radiation, which, according to this parameter, should result in higher mean sound volume. Modification in hot oil therefore contributes to improved acoustic parameters, measured dynamically, by measuring the time of passage of the sound wave, in large elements, while simultaneously affecting the same parameters determined in the static test.

## 4. Conclusions

On the basis of these results, it can be concluded that thermal modification of OHT adversely affects the acoustic parameters of small wooden elements. In contrast, for large elements (whole resonant wedges) OHT improved the acoustic parameters of resonant spruce wood. The ambiguous effect of OHT modification on different sample sizes results from differences in the absorption of the heating medium. The small samples absorbed relatively more oil, while increasing their density. Absorption took place at a depth of up to 2 mm, so in samples with a large cross-section, the effect of modification was an increase in acoustic properties. Sonic MOE values also increased in association with an increase in sound resistance. In the case of a resonant body, this means that more energy must be transferred from the strings to the body, in order to induce the plates to vibrate. However, it should be added that the speed of sound in the wood also improved, meaning that the wave propagation through the elements of the body is faster. It was further demonstrated that modification temperature does not affect the intensity of changes in the parameters of small wooden elements.

## Figures and Tables

**Table 1:** Mean value of spruce wood density before and after modification, at different temperatures and moisture content; ±(SD).

Modification Temperature	Oven-Dry			Air-Dry		
	*ρ*	*ρ_OHT_*	Δ*ρ*	*ρ*	*ρ_OHT_*	Δ*ρ*
(°C)	(kg × m^−3^)		(%)	(kg × m^−3^)		(%)
140	433 ± 57.6	476 ± 58.0	+9.9	476 ± 54.9	495 ± 53.7	+4.0
160	436 ± 56.5	471 ± 58.1	+8.0	475 ± 55.2	495 ± 60.9	+4.2
180	437 ± 59.1	468 ± 56.7	+7.1	471 ± 56.1	490 ± 57.2	+4.0
200	441 ± 57.5	472 ± 58.3	+7.0	478 ± 57.9	491 ± 58.0	+2.7

**Table 2:** Mean value of spruce wood modulus of elasticity (MOE), before to and after modifications, at different temperatures and moisture content levels; ±(SD).

Modification Temperature	Oven-Dry			Air-Dry		
	MOE	MOE_OHT_	ΔMOE	MOE	MOE_OHT_	ΔMOE
(°C)	(MPa)		(%)	(MPa)		(%)
140	11,550 ± 2043	12,064 ± 2101	+4.4	10,319 ± 1922	11,101 ± 2014	+7.6
160	11,755 ± 1984	12,072 ± 1757	+2.7	10,402 ± 2002	11,306 ± 2061	+8.7
180	11,709 ± 1935	12,173 ± 2032	+4.0	10,379 ± 1941	11,404 ± 2089	+9.9
200	11,716 ± 1995	12,144 ± 2049	+3.7	10,406 ± 1899	11,393 ± 2063	+9.5

**Table 3:** Mean value of spruce wood acoustic impedance, before and after modifications, at different temperatures and moisture content; ±(SD).

Modification Temperature	Oven-Dry			Air-Dry		
	*z*	*z_OHT_*	Δ*z*	*z*	*z_OHT_*	Δ*z*
(°C)	(kg × m^−2^ × s^−1^)		(%)	(kg × m^−2^ × s^−1^)		(%)
140	22.36 ± 0.344	23.96 ± 0.351	+7.2	22.16 ± 0.312	23.44 ± 0.342	+5.8
160	22.64 ± 0.334	23.85 ± 0.324	+5.3	22.23 ± 0.319	23.66 ± 0.338	+6.4
180	22.62 ± 0.353	23.87 ± 0.358	+5.5	22.11 ± 0.331	23.64 ± 0.329	+6.9
200	22.73 ± 0.338	23.94 ± 0.344	+5.3	22.30 ± 0.329	23.65 ± 0.317	+6.1

**Table 4:** Mean value of spruce wood acoustic constant, before and after modification, at different temperatures and moisture contents; ±(SD).

Modification Temperature	Oven-Dry			Air-Dry		
	*A*	*A_OHT_*	*A_OHT_/A*	*A*	*A_OHT_*	*A_OHT_/A*
(°C)	(m^4^ × kg^−1^ × s^−1^)		(%)	(m^4^ × kg^−1^ × s^−1^)		(%)
140	11.93 ± 1.314	10.57 ± 1.024	88.6	9.78 ± 1.008	9.57 ± 1.044	97.8
160	11.91 ± 1.300	10.78 ± 1.144	90.5	9.85 ± 0.992	9.65 ± 1.096	98.0
180	11.85 ± 1.342	10.89 ± 1.028	91.9	9.97 ± 1.017	9.85 ± 1.101	98.8
200	11.69 ± 1.309	10.75 ± 1.137	91.9	9.76 ± 1.089	9.81 ± 1.062	100.5

**Table 5:** Mean values of spruce-wood dynamic MOE, before and after modifications.

Sample Number	MOE_D_	MOE_DOHT_	ΔMOE_D_	*A*	*A_OHT_*	Δ*A*
	(MPa)		(%)	(m^4^ × kg^−1^ × s^−1^)		(%)
1	12,322	13,687	+11.1	12.85	13.35	+3.9
2	12,704	14,485	+14.0	14.58	15.75	+8.0
3	13,485	14,776	+9.6	12.00	12.57	+4.7
4	12,239	13,376	+9.3	10.59	11.07	+4.6
